# Association of State Policies Allowing Medical Cannabis for Opioid Use Disorder With Dispensary Marketing for This Indication

**DOI:** 10.1001/jamanetworkopen.2020.10001

**Published:** 2020-07-14

**Authors:** Chelsea L. Shover, Noel A. Vest, Derek Chen, Amanda Stueber, Titilola O. Falasinnu, Jennifer M. Hah, Jinhee Kim, Ian Mackey, Kenneth A. Weber, Maisa Ziadni, Keith Humphreys

**Affiliations:** 1 Department of Psychiatry and Behavioral Sciences, Stanford University School of Medicine, Stanford, California; 2 Department of Anesthesiology, Perioperative, and Pain Medicine, Stanford University School of Medicine, Stanford, California; 3 Department of Psychology, Washington State University, Seattle; 4 Department of Epidemiology and Population Sciences, Stanford University School of Medicine, Stanford, California; 5 Veterans Affairs Palo Alto Health Care System, Palo Alto, California

## Abstract

**Importance:**

Misinformation about cannabis and opioid use disorder (OUD) may increase morbidity and mortality if it leads individuals with OUD to forego evidence-based treatment. It has not been systematically evaluated whether officially designating OUD as a qualifying condition for medical cannabis is associated with cannabis dispensaries suggesting cannabis as a treatment for OUD.

**Objective:**

To examine whether state-level policies designating OUD a qualifying condition for medical cannabis are associated with more dispensaries claiming cannabis can treat OUD.

**Design, Setting, and Participants:**

This cross-sectional, mixed-methods study of 208 medical dispensary brands was conducted in 2019 using the brands’ online content. The study included dispensaries operating in New Jersey, New York, and Pennsylvania, where OUD is a qualifying condition for medical cannabis, and in Connecticut, Delaware, Maryland, Ohio, and West Virginia, where this policy does not exist.

**Exposures:**

Presence of OUD on the list of qualifying conditions for a state’s medical cannabis program.

**Main Outcomes and Measures:**

Binary indicators of whether online content from the brand said cannabis can treat OUD, can replace US Food and Drug Administration–approved medications for OUD, can be an adjunctive therapy to Food and Drug Administration–approved medications for OUD, or can be used as a substitute for opioids to treat other conditions (eg, chronic pain).

**Results:**

After excluding duplicates, listings for nonexistent dispensaries, and those without online content, 167 brands across 7 states were included in the analysis (44 [26.3%] in states where OUD was a qualifying condition and 123 [73.7%] in adjacent states). A dispensary listed in a directory for West Virginia was not operational; therefore, comparison states were Connecticut, Delaware, Maryland, and Ohio. In policy-exposed states, 39% (95% CI, 23%-55%) more dispensaries claimed cannabis could treat OUD compared with unexposed states (*P* < .001). For replacing medications for OUD and being an adjunctive therapy, the differences were 14% (95% CI, 2%-26%; *P* = .002) and 28% (95% CI, 14%-42%; *P* < .001), respectively. The suggestion that cannabis could substitute for opioids (eg, to treat chronic pain) was made by 25% (95% CI, 9%-41%) more brands in policy-exposed states than adjacent states (*P* = .002).

**Conclusions and Relevance:**

In this study, state-level policies designating OUD as a qualifying condition for medical cannabis were associated with more dispensaries claiming cannabis can treat OUD. In the current policy environment, in which medical claims by cannabis dispensaries are largely unregulated, these advertisements could harm patients. Future research linking these policies to patient outcomes is warranted.

## Key Points

**Question:**

Is making opioid use disorder (OUD) a qualifying condition for medical cannabis associated with dispensaries promoting cannabis as a treatment for OUD?

**Findings:**

In this cross-sectional study of the online content of 167 medical cannabis dispensaries, compared with dispensaries in states where OUD was not a qualifying condition for medical cannabis, 39% more dispensaries in states where this policy was enacted promoted cannabis to treat OUD and 14% more recommended replacing US Food and Drug Administration–approved medications for OUD with cannabis.

**Meaning:**

In this study, officially designating OUD a qualifying condition for medical cannabis was associated with cannabis dispensaries making unsupported medical claims regarding using cannabis to treat OUD.

## Teaser

This cross-sectional study examines whether state-level policies designating opioid use disorder (OUD) a qualifying condition for medical cannabis are associated with dispensaries claiming that cannabis can treat OUD.

## Introduction

Many producers and sellers of medical cannabis products make unsubstantiated claims about therapeutic benefits.^1,2^ Unsupported therapeutic claims carry particular risks in the context of the US epidemic of opioid use disorder (OUD), which causes approximately 50 000 deaths annually.^2^ Specifically, some medical cannabis sellers encourage people with OUD to replace US Food and Drug Administration (FDA)–approved medications for OUD (MOUDs) with cannabis. Abundant evidence supports the safety and efficacy of FDA-approved MOUDs (ie, methadone, buprenorphine, naltrexone), and individuals who do not take them more than double their risk of mortality.^3^

The claim that cannabis can treat OUD is often touted via the cannabis industry’s research as marketing. For example, the company Weedmaps leased billboards reading, “States that legalized medical marijuana had 25% fewer opioid-related deaths.”^4^ These advertisements usually cite an early study showing that through 2010, legalization of medical cannabis was negatively correlated at the state level with opioid overdoses.^4,5^ However, a study using identical methods through 2017 found that this correlation had turned positive.^6^ More generally, never in the history of medicine has a drug been approved to treat a serious condition based on ecological correlations in uncontrolled research.^2^

Nevertheless, several states have sanctioned OUD as an indication to receive medical cannabis.^7,8,9,10^ Whether these policies are associated with cannabis dispensaries promoting cannabis as a treatment for OUD or as a replacement for FDA-approved MOUDs is not known, nor has the prevalence of unsupported therapeutic claims been evaluated more generally.

To investigate the association between OUD being officially designated as a qualifying condition for medical cannabis and dispensary claims related to OUD, we conducted a rapid online mixed-methods study^11^ of dispensary advertisements in the 3 following northeastern states: New Jersey, New York, and Pennsylvania. We designated the 5 adjacent states that had medical cannabis programs without recreational programs as comparison states, as follows: Connecticut, Delaware, Maryland, Ohio, and West Virginia. We hypothesized that more medical cannabis dispensary brands in states where OUD was a qualifying condition would claim that cannabis can treat OUD than brands in states without this policy.

## Methods

This research was designated as exempt from review by Stanford University’s institutional review board. The study followed the Strengthening the Reporting of Observational Studies in Epidemiology (STROBE) reporting guideline. A list of unique dispensary companies (hereafter brands) was constructed for each state from state Department of Public Health websites and 4 industry websites previously identified in the literature.^12^ To validate initial keywords (*opioid*, *addiction*, *methadone*, *buprenorphine*, *suboxone*), 2 of us (C.L.S. and N.A.V.) independently reviewed website and social media accounts for a 5% sample (weighted for number of brands in a state) to assess whether keywords sufficiently captured relevant communications. After the pilot, *opioids* was added, and it was noted that Facebook’s internal search tool missed some posts containing the keywords. Scope of information bias because of missing posts was estimated by bulk-extracting Facebook data using Khoros Intelligence (Khoros) and coding the text. This additional data would have changed 5 (<1%) coded variables.

**Figure 1.**
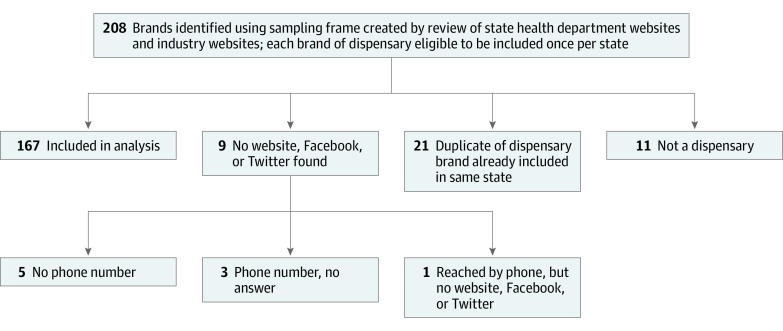
Diagram of Dispensary Brands Included in the Analysis State health department websites were reviewed for Connecticut, Delaware, Maryland, New Jersey, New York, Pennsylvania, Ohio, and West Virginia. Industry websites included Weedmaps, Leafly, Wheresweed.com, and Stickyguide.

For the full review in October 2019, 2 coauthors per brand independently searched for website and social media accounts; mentions of the 6 keywords were recorded using Qualtrics (SAP). If no online accounts were found but a phone number was listed in 1 of the directories, a coauthor called to obtain the website (Figure 1).

### Statistical Analysis

Collected text was independently coded by 3 of us (C.L.S., N.A.V., and J.M.H.) for mention of opioids and 4 binary indicators, as follows: did the brand claim cannabis can (1) treat OUD, (2) replace FDA-approved MOUDs, (3) be an adjunct to FDA-approved MOUDs, or (4) substitute for opioids to treat other conditions (eg, chronic pain). Two coders reviewed each dispensary; ratings were averaged to improve validity.^13^ Reliability was high (pooled κ = 0.85, with 93% agreement across coded variables).^14^

Difference in proportions of brands with each criterion were calculated and compared using a 1-tailed *Z* test (α = .05) to identify whether the claims were more commonly made by brands in policy-exposed states. As a post hoc analysis, we investigated whether differences by policy status were observable within brands that operated in both policy-exposed and policy-unexposed states. Analyses were conducted using Stata version 16 (StataCorp).

## Results

A total of 208 brands were identified. After removing duplicates, listings for nonexistent dispensaries, and those without online content, 167 medical dispensary brands in 7 states were identified, 44 (26.3%) in states where OUD was a qualifying condition and 123 (73.7%) in adjacent states. A dispensary listed in a directory for West Virginia was not operational; therefore, comparison states included Connecticut, Delaware, Maryland, and Ohio.

**Figure 2.**
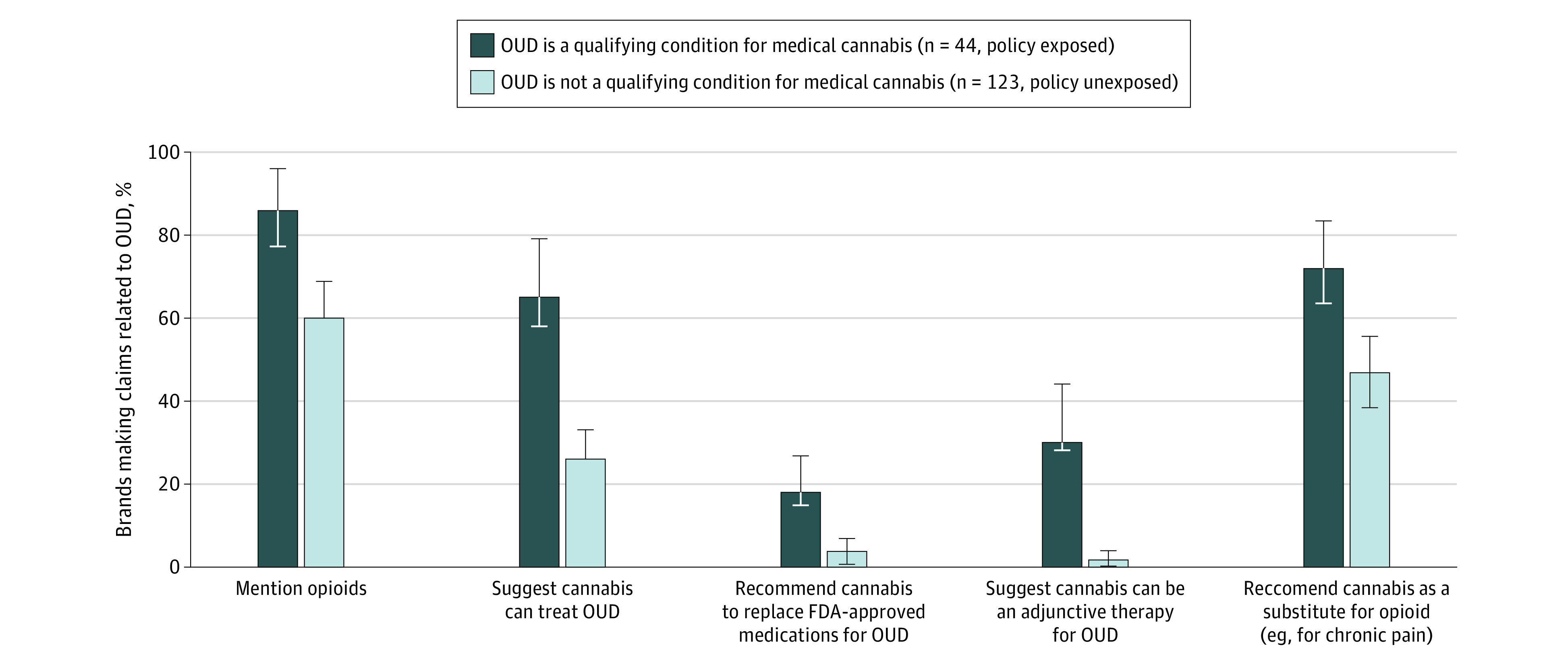
Proportion of Medical Cannabis Dispensary Brands Making Claims Related to Opioid Use Disorder (OUD) by Policy Status Whiskers indicate 95% CIs. FDA indicates US Food and Drug Administration.

All claims were significantly more common in states with OUD as a qualifying condition than in adjacent states (Figure 2). Regardless of policy status, most dispensary brands mentioned opioids, though 26% (95% CI, 13%-39%) more did so in New Jersey, New York, and Pennsylvania compared with adjacent states (*P* < .001). In policy-exposed states, 39% (95% CI, 23%-55%) more brands suggested that cannabis could treat OUD compared with brands in policy-unexposed states (*P* < .001). Recommendations to replace FDA-approved MOUDs with cannabis were less common overall, with 14% (95% CI 2%-26%) more brands making this claim in policy-exposed states than in adjacent states (*P* = .001). Cannabis was recommended as an adjunctive treatment for MOUDs by 28% (95% CI 14%-42%) more dispensary brands in policy-exposed states than in adjacent states (*P* < .001). The suggestion that cannabis could substitute for opioids (eg, to treat chronic pain) was made by 25% (95% CI, 9%-41%) more brands in policy-exposed states than in adjacent states (*P* = .002). Excerpts from dispensary brands that suggest cannabis can treat OUD, replace FDA-approved MOUDs, be used as an adjunctive therapy, or be used as a substitute for opioids are displayed in the Table.

**Table.** Excerpts From Medical Cannabis Dispensary Websites and Social Media Accounts Regarding Opioids

Policy status and state	Quote
**Suggest cannabis can treat OUD**	
Exposed, New York	“We care about you and your health. Join the discussion on how medical cannabis can treat and relieve opioid usage”
Unexposed, Maryland	“#Cannabis is NOT a gateway drug. It is a good way to help get off #Opioids”
Exposed, Pennsylvania	“Another benefit of CBD treatment being non–habit forming is that they can be a great option for curbing the effects of addiction. CBD has the potential to diminish the effects of opioid cravings and treat the side effects of drug withdrawals such as anxiety, restlessness, and nausea”
**Recommend cannabis to replace FDA-approved medications for OUD**	
Unexposed, Maryland	“The fact is, cannabis poses less of a risk than current FDA-approved opioid-based treatments like methadone. Patients see better treatment outcomes when they have access to cannabis, and many health care providers have seen high-dose opiate patients significantly reduce or eliminate opiates with the use of cannabis. So why isn’t Maryland using cannabis is fight OUD?”
Exposed, Pennsylvania	“In dealing with opioid addiction, cannabis can be safer than other harm reduction options like methadone and Suboxone. It does not have the risk of a fatal overdose and has a lower risk of dependence and problematic use than other psychoactive substances. Cannabis can be used in combination with methadone or Suboxone to enhance the benefits and support a taper of these drugs”
Exposed, Pennsylvania	“In the long run, medical marijuana might turn into another form of medication-assisted treatment (known as MAT), like methadone and Suboxone, which use milder opioids to help people get off more dangerous ones. Today, MAT is in short supply, with fewer than one-third of the people who want such treatment getting it. Cannabis, in contrast, is now legal in 32 states. So the expansion of medical marijuana dispensaries nationwide might offer one avenue for offering the treatment to a wider audience”
**Suggest cannabis can be an adjunctive therapy for OUD**	
Exposed, New Jersey	“Approved debilitating medical conditions include: Amyotrophic lateral sclerosis, Anxiety, Chronic pain related to musculoskeletal disorders, Chronic pain of visceral origin, Migraine, Multiple sclerosis, Opioid Use Disorder as an adjunct to Medication Assisted Therapy, Terminal cancer, Muscular dystrophy, Inflammatory bowel disease, including Crohn’s disease, Terminal illness, if the physician has determined a prognosis of less than 12 months of life, Tourette’s Syndrome”
Unexposed, Maryland	“Cannabis usage may increase the likelihood of the completion of opioid treatment programs”
Exposed, Pennsylvania	“Opioid use disorder for which conventional therapeutic interventions are contraindicated or ineffective, or for which adjunctive therapy is indicated in combination with primary therapeutic interventions”
**Recommend cannabis as a substitute for opioids, eg, to treat chronic pain**	
Exposed, Pennsylvania	“Cannabis has been proven to be much better at treating most chronic pain patients than opioids and is much safer”
Unexposed, Ohio	“Medical marijuana in States where it is legal may serve as a less harmful alternative to opioids in treating veterans”
Unexposed, Connecticut	“We are in the throes of an opioid abuse crisis and are desperately searching for an answer. It’s time we acknowledge the solution that’s right in front of us and make this life-saving treatment available for those dependent on opioids. Cannabis has been proven to relieve chronic pain while reducing and replacing the use of opioids. It also relieves the symptoms of opioid withdrawal and decreases opioid craving. There is no toxic or lethal overdose of cannabis, and thousands of patients in Maine are already effectively using cannabis to replace opioids and other addictive substances”

Abbreviations: CBD, cannabidiol; FDA, US Food and Drug Administration; OUD, opioid use disorder.

Overall, 14 brands (8.4%) operated in more than 1 state; of these, 11 (78.6%) operated in both policy-exposed and policy-unexposed states. Within these 11 brands, no significant differences in any of the outcomes were observed by policy status, although this post hoc analysis included only 29 observations.

## Discussion

The results of this study demonstrated that a large proportion of dispensaries make unsupported claims regarding the effectiveness of cannabis as a treatment for OUD, including that cannabis should replace FDA-approved MOUDs. These claims are more prevalent in states where regulators have designated OUD as a qualifying condition to access medical cannabis. Public health officials who enacted these policies likely did not intend to drive people with OUD away from effective treatments. However, that is what the dispensaries’ medical claims could do if these communications influence patients’ OUD treatment decisions. Addiction medicine professionals have previously raised concerns regarding policies that add opioid use or OUD as a qualifying condition for medical cannabis.^2^ To our knowledge, this study provides the first empirical evidence of an association between these policies and unsubstantiated claims related to treating OUD. Policy makers weighing similar changes should take this as a caution.

The low bar of evidence set for endorsing a putative addiction treatment may reflect OUD’s stigmatized status.^2^ However, asking medical cannabis dispensaries to uphold the safety and efficacy standards normally associated with medicine is not an issue specific to OUD. Medical cannabis is promoted as a treatment for Alzheimer disease, cancer, depression, diabetes, heart disease, obesity, Parkinson disease, and more.^4,15^ This study uniquely demonstrates the association between variations in medical cannabis policies and the prevalence of unsupported medical claims. Future research should elucidate whether and how these claims adversely affect patients’ health.

Although data linking medical cannabis dispensary advertisements and patient decision-making about opioids and OUD treatment are beyond the scope of the current study, relevant evidence from similar lines of inquiry demonstrates the influence of advertisements on consumer behavior. Cannabis dispensary advertising generally has been shown to be associated with increased use among adolescents.^16,17^ Robust literature on the marketing of products or behaviors that can affect health (eg, public health messages, selling alcohol or tobacco, and direct-to-consumer advertising of pharmaceuticals) consistently shows at least modest associations between communications from brands and consumer or patient behavior.^18,19,20,21^ Therefore, the findings of this study underscore the need to evaluate whether and how cannabis dispensaries’ medical messaging influences patient behavior and decision-making regarding OUD.

Regulatory action against unsupported therapeutic claims by the medical cannabis industry has thus far been anemic. As of January 21, 2020, only 1 brand included in this study had received a warning letter from the FDA regarding unapproved and misbranded human drug products.^15^ This July 2019 letter cited the following example of an unsupported medical claim: “Cannabidiol [CBD] can also be used in conjunction with opioid medications, and a number of studies have demonstrated that CBD can in fact reduce the severity of opioid-related withdrawal and lessen the buildup of tolerance.” Our study, which was conducted 3 months later, found that the company still had online content with similar claims. Enforcement is warranted. In the meantime, policy makers should ask why the medical cannabis industry is not being held to even minimal medical standards regarding claims about treating serious medical conditions.^22^

### Limitations

This study has limitations. A study of online promotions will understate the prevalence of unsupported therapeutic claims if such claims are only made within the physical environment of the dispensary. Because each brand was included once per state, if a dispensary operated multiple social media accounts for different locations within the state, the content could differ, but this misclassification seems unlikely to differ by policy status. Most website content was undated, so it was impossible to determine whether that content preceded the addition of OUD as a qualifying condition for medical cannabis. Posts missed owing to errors in Facebook’s internal search tool could bias proportions downward, although we expect this to be minimal based on the additional Khoros reports.

## Conclusions

In this study, state-level policies designating OUD as a qualifying condition for medical cannabis were associated with more dispensaries claiming cannabis can treat OUD. Future studies linking dispensary advertisements to health outcomes in people with OUD (eg, use of MOUDs, overdose mortality) are warranted, as are investigations into advertising that addresses other conditions with major morbidity and mortality consequences. Understanding the degree to which marketing by cannabis dispensaries influences patient decision-making related to OUD treatment is also important, particularly in the context of different state-level cannabis policies. It will additionally be informative to evaluate whether states that legalize recreational cannabis continue to have medical advertising content.
